# Income Inequalities in Hepatitis B Vaccination and Willingness to Pay Among Women of Reproductive Age in Hanoi, Vietnam

**DOI:** 10.9745/GHSP-D-20-00480

**Published:** 2021-09-30

**Authors:** Xuan Thi Thanh Le, Nguyen Thao Thi Nguyen, Huong Thi Le, Toan Thanh Thi Do, Thang Huu Nguyen, Huong Lan Thi Nguyen, Trang Ha Nguyen, Linh Gia Vu, Bach Xuan Tran, Carl A. Latkin, Cyrus S.H. Ho, Roger C.M. Ho

**Affiliations:** a School for Preventive Medicine and Public Health, Hanoi Medical University, Hanoi, Vietnam.; b Duke University School of Medicine, Duke University, Durham, NC, USA.; c Institute for Global Health Innovations, Duy Tan University, Da Nang, Vietnam.; d Faculty of Nursing, Duy Tan University, Da Nang, Vietnam.; e Center of Excellence in Evidence-based Medicine, Nguyen Tat Thanh University, Ho Chi Minh City, Vietnam.; f Johns Hopkins Bloomberg School of Public Health, Baltimore, MD, USA.; g Department of Psychological Medicine, National University Hospital, Singapore, Singapore.; h Department of Psychological Medicine, Yong Loo Lin School of Medicine, National University of Singapore, Singapore, Singapore.; i Institute for Health Innovation and Technology (iHealthtech), National University of Singapore, Singapore, Singapore.

## Abstract

**Background:**

Hepatitis B virus (HBV) infection is endemic in Vietnam and can be transmitted from mother to child. Vaccination of women of reproductive age (WRA) can reduce this transmission. Because adult HBV vaccination in Vietnam follows a fee-for-service model, research is needed to determine the effect of household income on willingness to pay (WTP) to ensure equitable access to the vaccine.

**Methods:**

A cross-sectional study was performed in Hanoi, Vietnam, in April 2018, among WRA. Questionnaires were administered to assess household income, HBV history, vaccination status, vaccine awareness, and WTP for the vaccine. Multivariable logistic and interval regression were performed to assess the impact of household income on WTP for HBV vaccine.

**Results:**

This study found that 62.3% of all participants were willing to pay for the HBV vaccine with no differences in WTP across income quintiles. There were significant differences among household income levels in awareness of HBV vaccination and WTP amount beyond US$4.50 with the lowest awareness and WTP amount among women from the lowest income quintiles.

**Conclusions:**

Our data suggest the need to subsidize HBV vaccination for low-income women to ensure more equitable access to HBV vaccination. We propose that a sliding-scale payment method may be an effective strategy in light of limited funding to support vaccination expansion. An education campaign focusing on lower-income households should also be implemented in conjunction with this program. Further research would be required to evaluate consumer acceptance of this payment scheme and to develop an appropriate sliding scale to maximize vaccine uptake.

## Abstract (precis)

Many countries use a fee-for-service model for hepatitis B vaccination, which amplifies health disparities across socioeconomic statuses and contributes to inequalities in HBV vaccination rates. We examined the role of household income on women’s willingness to pay and the amount they are willing to pay for HBV vaccination to identify a more optimized payment scheme and equitable access across all income groups.

## Teaser

**Key Findings:**

- Most women were willing to pay for the hepatitis B virus (HBV) vaccine and there was no difference in willingness to pay for HBV vaccination between income quintiles.
- The amount willing to pay for the vaccine was different between income levels for prices higher than US$4.50.

**Key Implications:**

- Because women from the lowest income quintile had the lowest awareness of the HBV vaccine, efforts on education and awareness of HBV and the vaccine should focus on low-income women.
- Given the high vertical transmission rates of HBV infection, policy makers should prioritize vaccinating women of reproductive age as well as newborns.
- In light of the finding that higher-income women were willing to pay more for the vaccine, adopting a sliding-scale payment system may mitigate challenges with funding and provide opportunities for equitable vaccine access.

## INTRODUCTION

Hepatitis B virus (HBV) infection can cause both acute and chronic liver disease. In a small percentage of patients whose immune systems are unable to clear the virus, particularly children aged younger than 6 years old, HBV infection becomes chronic, often leading to cirrhosis and hepatocellular carcinoma.^1^ Though increasing availability of HBV vaccination has decreased morbidity and mortality, as of 2015, the World Health Organization (WHO) estimated that 257 million people worldwide are still living with chronic HBV infection with approximately 887,000 annual deaths due to cirrhosis or hepatocellular carcinoma.^1^

Vietnam has one of the highest rates of HBV infection in the world with 8.8%–19.0% of the general population estimated to be hepatitis B surface antigen-positive (HBsAg-positive).^2^^–^^5^ The disease is primarily vertically transmitted from mother to child during labor or less commonly, in-utero.^6^^,^^7^ If infants do not receive HBV immunoglobulin and vaccination at birth, mothers who are HBsAg-positive may transmit HBV infection at rates up to 90%.^8^ It is estimated that at least 50% of HBV-positive individuals acquired their infection perinatally or in early childhood.^9^ Horizontal transmission of HBV and disease incidence in adulthood is less clearly studied though data have suggested that extrafamilial horizontal transmission is likely high.^10^

Especially in endemic countries, HBV vaccination of neonates is an effective strategy to decrease transmission.^11^ In 2006, Vietnam began implementing widespread neonatal vaccination, resulting in a decrease in children who were HBsAg-positive from 3.62% to less than 2% between study periods 2000–2003 and 2008–2011.^12^ However, a national study in 2014 found that only 62.8% of children received the birth dose—far from universal coverage.^12^ As such, additional approaches should be considered to further decrease transmission rates.

A promising strategy that has been proposed to decrease rates of HBV infection transmission and improve disease control is vaccination of women of reproductive age (WRA).^13^ During pregnancy, women are vaccinated to confer immunity to neonates,^14^ given the risk of horizontal transmission in adulthood. However, there is a lack of randomized controlled trials on the efficacy of HBV vaccination for WRA to prevent maternal infection and consequently neonatal infection.

Currently, HBV vaccination among adults in Vietnam follows a fee-for-service model, which has worsened health disparities across socioeconomic statuses.^15^ Indeed, in other countries that follow a similar model, including China and South Korea, income was found to be the largest contributor to inequalities in HBV vaccination.^16^^,^^17^ Studies examining the effect of income on HBV vaccination have not been conducted in Vietnam. This study aims to fill this gap by elucidating the role of household income on WRA’s willingness to pay (WTP) and the amount they are willing to pay for HBV vaccination in an effort to pursue a more optimized payment scheme and equitable access across all income groups.

HBV vaccination among adults in Vietnam follows a fee-for-service model, which has worsened health disparities across socioeconomic statuses.

## METHODS

### Study Design and Sample

We performed a cross-sectional study in Dong Da (urban setting) and Ba Vi (rural setting) districts, Hanoi, Vietnam, in April 2018. In each district, we randomly selected 2 communes—Trung Tu and Phuong Lien communes in Dong Da district and Thuy An and Phong Van communes in Ba Vi district.

Women were invited to participate in this study if they were pregnant or had a child aged younger than 12 months. Other inclusion criteria were residence in the study setting for at least 6 months and willingness to participate in the study. Women were excluded if they had any cognitive impairment or disabilities that might affect their ability to understand and answer the questionnaire. A list of all eligible women in the study sites was compiled with the support of local health authorities. Then, participants were randomly selected using computer software and contacted via phone. If they refused to participate, we invited the next individual on the list. A total of 764 women were contacted to enroll in the study, and no one refused. However, data of only 695 women were included in the study because some participants did not report monthly household income (response rate 91.0%).

### Ethics Approval

The protocol of this study was reviewed and approved by the Ethics Committee of Hanoi Medical University (Code number:184/HMU-IRB; November 14, 2015). After hearing a one-on-one explanation of the study by trained health care workers at the Hanoi Medical University, all participants gave their verbal informed consent before participating in the study, acknowledging full understanding of the study’s purpose, their rights to withdraw from the study at any time, and protection of confidentiality.

### Data Collection and Measurement

**FIGURE 1:**
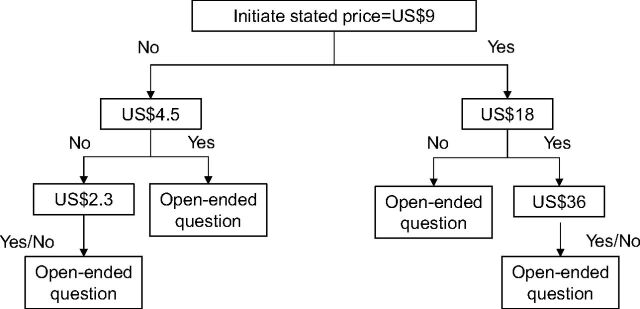
Bidding Process Used During Interviews With Women of Reproductive Age to Determine Willingness to Pay for One Dose of Hepatitis B Vaccine, Hanoi, Vietnam

Face-to-face interviews were conducted by medical students and health care workers at Hanoi Medical University in 2018. These data collectors were trained extensively regarding study purpose, communication, and interview skills. Moreover, they participated in piloting the structured questionnaire to ensure the consistency of the data collection process. Each interview lasted 20–25 minutes. The questionnaire included questions regarding the following:

- Sociodemographic characteristics. We asked participants to report their age, education level, occupation, number of children, and residential setting (urban/rural). Household economic status was divided into 5 quintiles based on total household monthly income.
- HBV history, vaccination awareness, and uptake. We collected information on history of HBV infection, awareness of HBV vaccine, source of general vaccination knowledge, vaccination status against HBV, and willingness to pay for HBV vaccines in the future.
- Willingness to pay for HBV vaccine. We applied a contingent valuation approach through double-bounded dichotomous choice to elicit WTP and the amount participants were willing to pay for 1 dose of HBV vaccine. The bidding process is illustrated in Figure 1. We first informed the women about the HBV vaccine and its effects on HBV prevention. Then, we asked them to state their willingness to pay for the vaccine. We used 200,000 VND (approximately US$9) for a single vaccine as a first bid. This price was selected based on the actual price for the on-demand vaccination service. Initially, we asked participants about their WTP for the first bid. If they answered “no,” they were asked whether they were willing to pay US$4.50. If they had answered “yes,” to the first bid, they were asked about their willingness to pay US$18. At the end of the process, participants were asked the maximum amount they were willing to pay for 1 dose of the HBV vaccine.

### Statistical Analysis

The data were analyzed by STATA version 14.0. Chi-squared and Kruska-Wallis tests were used to examine the difference in various characteristics among 5 household income quintiles. The differences in WTP by income quintile were explored by plotting the percentage of each quintile that was willing to pay a particular value or higher. As the value increases, the curves fall to reflect decreasing proportions of participants willing to pay higher prices. Lorenz curves and Gini coefficients were used to measure the extent of inequality based on the history of HBV vaccination and WTP for the HBV vaccine.

Multivariable logistic and interval regression were then performed to examine the factors associated with WTP and the maximum amount participants were willing to pay for 1 dose of HBV vaccine. These regression techniques were used along with stepwise forward selection strategies. A *P*-value <.2 was employed for variable selection. Statistical significance was determined if the *P*-value <.05.

## RESULTS

Table 1 lists these findings with Q1 corresponding to the quintile with the lowest income and Q5 the highest income. Of 695 participants, the mean age and household monthly income were 27 years and US$663.20, respectively. Household monthly income, residential setting (urban versus rural), and education level were significantly different between income quintiles (*P*<.05). The lowest quintile had the largest percentage of rural residents, as well as the largest percentage of participants with high school and lower levels of education.

**TABLE 1.** Sociodemographic Characteristics of Participants in Study of Effects of Household Income on Willingness to Pay for Hepatitis B Vaccine, Hanoi, Vietnam, (n=695)

	**Income Quintiles**						
	**Q1**	**Q2**	**Q3**	**Q4**	**Q5**	**Total**	***P* Value **
Total, n (%)	124 (17.8)	211 (30.4)	131 (18.9)	120 (17.3)	109 (15.7)	695 (100.0)	
Mean age, years	26.8	26.2	28.1	27.6	27.1	27.0	.06
Mean household monthly income, US$	187.0	389.6	611.6	830.0	1612.8	663.2	<.01^a^
Married, n (%)	120 (96.8)	204 (97.1)	130 (100.0)	117 (98.3)	108 (100.0)	679 (98.3)	.12
Living in rural area, n (%)	107 (90.7)	151 (72.6)	42 (32.6)	25 (21.4)	14 (12.8)	339 (49.8)	<.01^a^
Having any children, n (%)	119 (96.0)	192 (91.0)	119 (90.8)	110 (91.7)	101 (92.7)	641 (92.2)	.51
Education, n (%)							
≤ Secondary	48 (38.7)	29 (13.7)	10 (7.6)	5 (4.2)	2 (1.8)	94 (13.5)	<.01^a^
High	51 (41.1)	85 (40.3)	40 (30.5)	20 (16.7)	19 (17.4)	215 (30.9)	
Vocational training	13 (10.5)	52 (24.6)	27 (20.6)	31 (25.8)	16 (14.7)	139 (20.0)	
University	12 (9.7)	45 (21.3)	54 (41.2)	64 (53.3)	72 (66.1)	247 (35.5)	

^a^ Significant at *P*<.05.

Table 2 shows that 3.2% of participants had a history of HBV infection. The percentage of women who were aware of the HBV vaccine was significantly different across quintiles with the lowest rates in Q1 (86.3%) and the highest in Q5 (98.2%) (*P*<0.5). Moreover, the percentage of women who had previously received the HBV vaccine was significantly different between quintiles with the lowest rates in Q1 (18.6%) and higher rates in Q5 (40.4%) (*P*<0.05). Only 1.1% injected 3 doses of vaccine during the pregnancy. People with higher income were more likely to be injected with a higher number of doses (*P*<0.05). Among all quintiles, 62.3% of women were willing to pay for the HBV vaccine, with a mean maximum amount of US$10.30. No significant difference was found between quintile groups regarding WTP and the maximum amount.

**TABLE 2.** Hepatitis B Vaccination Awareness, Uptake, and Willingness to Pay Among Participants in Study of Effects of Household Income on Willingness to Pay for the Vaccine, Hanoi, Vietnam, (n=695)

**Characteristics**	**Income Quintiles**						
	**Q1**	**Q2**	**Q3**	**Q4**	**Q5**	**Total**	***P* Value **
**History of Hepatitis B virus infection, n (%)**	8 (6.5)	7 (3.3)	2 (2.3)	2 (1.7)	2 (1.8)	22 (3.2)	.19
**Awareness of Hepatitis B vaccine, n (%)**	107 (86.3)	202 (95.7)	121 (92.4)	116 (96.7)	107 (98.2)	653 (94.0)	<.01^a^
**Source of general vaccination information, n (%)**							
School	6 (4.8)	11 (5.2)	6 (4.6)	5 (4.2)	8 (7.3)	36 (5.2)	.84
Television	30 (24.2)	70 (33.2)	57 (43.5)	57 (47.5)	43 (39.5)	257 (37.0)	<.01^a^
Radio or loudspeaker	45 (36.3)	81 (38.4)	23 (17.6)	27 (22.5)	17 (15.6)	193 (27.8)	<.01^a^
Newspapers/magazines	16 (12.9)	36 (17.1)	41 (31.3)	38 (31.7)	28 (25.7)	159 (22.9)	<.01^a^
Internet	35 (28.2)	95 (45.0)	82 (62.6)	81 (67.5)	83 (76.2)	376 (54.1)	<.01^a^
Health workers	79 (63.7)	129 (61.1)	78 (59.5)	51 (42.5)	51 (46.8)	388 (55.8)	<.01^a^
Friends and relatives	6 (4.8)	18 (8.5)	32 (24.4)	23 (19.2)	30 (27.5)	109 (15.7)	<.01^a^
Other	8 (6.5)	13 (6.2)	2 (1.5)	5 (4.2)	2 (1.8)	30 (4.3)	.13
**History of Hepatitis B vaccination, n (%)**	23 (18.6)	71 (33.7)	58 (44.3)	53 (44.2)	44 (40.4)	249 (35.8)	<.01^a^
**Willingness to pay for Hepatitis B vaccine, n (%)**	78 (62.9)	123 (58.6)	81 (61.8)	80 (67.2)	70 (64.2)	432 (62.3)	.61
**Maximum amount willing to pay for Hepatitis B vaccine**							
Mean, US$	8.0	9.8	9.9	11.0	13.2	10.3	.09
Median, US$	5.3	6.4	8.5	8.5	8.5	8.5	.09

^a^ Significant at *P*<.05.

The percentage of women who were aware of the HBV vaccine was significantly different across quintiles.

**FIGURE 2:**
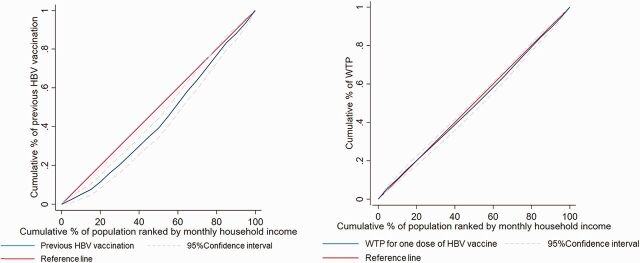
(a) Lorenz Curve Showing History of Hepatitis B Vaccination Among Women of Reproductive Age, Hanoi, Vietnam, by Monthly Household Income; (b) Lorenz Curve Showing Willingness to Pay for One Dose of Hepatitis B Vaccine Among Women of Reproductive Age, Hanoi, Vietnam, by Monthly Household Income Abbreviations: HBV, hepatitis B virus; WTP, willingness to pay.

The Lorenz curves in Figure 2 show that the distribution of previous HBV vaccination was not equalized (Gini coefficient=0.13) when household income was taken into account. On the other hand, the distribution of WTP for the HBV vaccine was approximately equalized (Gini coefficients=0.02) among the various household income levels.

**FIGURE 3:**
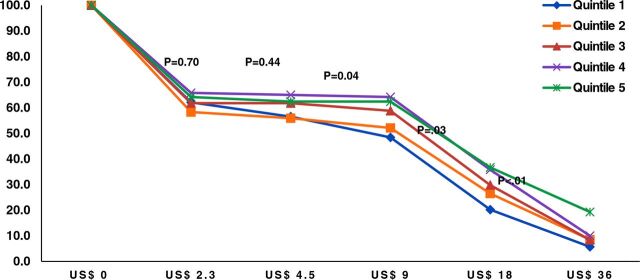
Cumulative Percentage of Women Willing to Pay Various Amounts for Hepatitis B Vaccine, Hanoi, Vietnam, by Household Income Quintile

Figure 3 shows the cumulative percentage of participants willing to pay various amounts for HBV vaccination by household income quintiles. Differences between Q1 and Q5 were significant at US$9, $18, and$36.

Table 3 shows that 37.7% of women were not willing to pay for the vaccine. The major reason was “not necessary” (45.2%), following by “no risk of HBV infection” (18.4%), “others” (17.2%), and “already injected” (16.5%). Significant differences between people who were and were not willing to pay for the vaccine were found in education levels; awareness of HBV vaccine; and using school, radio/loudspeaker, Internet, and friends/relatives as sources of information (*P*<.05).

**TABLE 3.** Characteristics of Participants Who Were and Were Not Willing to Pay for Hepatitis B Vaccines and Reasons for Not Willing to Pay

**Characteristics**	**Not Willing to Pay**	**Willing to Pay**	***P* Value **
**Total, n (%)**	261 (37.7)	432 (62.3)	
**Age, mean (SD), years**	27.2 (6.8)	27.0 (5.0)	.92
**Household monthly income, mean (SD), US$**	642.8 (550.9)	676.2 (548.6)	.36
**Married, n (%)**	258 (98.9)	419 (97.9)	.35
**Living in rural area, n (%)**	122 (48.8)	215 (50.1)	.74
**Having any children, n (%)**	242 (92.7)	399 (92.4)	.86
**Education, n (%)**			
≤ Secondary	45 (17.2)	49 (11.3)	.03
High	72 (27.6)	143 (33.1)	
Vocational training	43 (16.5)	94 (21.8)	
University	101 (38.7)	146 (33.8)	
**History of hepatitis B vaccine, n (%)**	10 (3.9)	12 (2.8)	.44
**Awareness of hepatitis B vaccine, n (%)**	239 (91.6)	413 (95.6)	.03
**Source of general vaccination information, n (%)**			
School	20 (7.7)	16 (3.7)	.02
Television	86 (33.0)	171 (39.6)	.08
Radio or loudspeaker	61 (23.4)	132 (30.6)	.04
Newspapers/magazines	57 (21.8)	102 (23.6)	.59
Internet	125 (47.9)	251 (58.1)	<.01
Health workers	134 (51.3)	253 (58.6)	.06
Friends and relatives	31 (11.9)	78 (18.1)	.03
Other	9 (3.5)	20 (4.6)	.45
**History of hepatitis B vaccination, n (%)**	91 (34.9)	158 (36.6)	.65
**Reasons for not being willing to pay**			
Not necessary	118 (45.2)		
Unaffordable	7 (2.7)		
No risk of hepatitis B virus infection	48 (18.4)		
Already injected	43 (16.5)		
Others	45 (17.2)		

Abbreviation: SD, standard deviation.

Table 4 reveals that income quintile groups (except Q2 for WTP) were not independent factors associated with previous HBV vaccination, WTP, and the amount willing to pay for HBV vaccine after adjusting for other confounders. Women with university degrees, without spouse/partner, who received vaccine information from newspapers/magazines, and who had heard about HBV vaccination were more likely to have been previously vaccinated against HBV. The source of vaccination information was found to be associated with WTP for the HBV vaccine. Education, marital status, and source of information were associated with the maximum amount participants were willing to pay.

**TABLE 4.** Factors Associated With Willingness to Pay for HBV Vaccine and Maximum Amount

**Characteristic**	**Previous HBV vaccination**		**WTP for HBV vaccine**		**Amount of WTP for one dose of HBV vaccine**	
	**OR**	**95%CI**	**OR**	**95%CI**	**Coef.**	**95%CI**
**Education**						
≤ Secondary	ref		ref		ref	
High	0.99	0.52, 1.88	1.55	0.89, 2.69	6.44^a^	1.95, 10.93
Vocational training	1.30	0.66, 2.58	1.51	0.81, 2.81	6.62^a^	1.64, 11.61
University	2.29^a^	1.16, 4.51	0.89	0.49, 1.62	3.17	-1.65, 7.99
**Household monthly income quintiles**						
Q1	ref		ref		ref	
Q2	1.73	0.97, 3.10	0.58^a^	0.35, 0.98	–1.14	-5.24, 2.96
Q3	1.87	0.96, 3.65	0.73	0.40, 1.32	0.36	-4.36, 5.08
Q4	1.56	0.78, 3.11	1.08	0.57, 2.05	1.87	-3.05, 6.80
Q5	1.28	0.62, 2.63	0.89	0.46, 1.70	3.93	-1.19, 9.05
**Marital status**						
Having spouse/partner	ref		ref		ref	
Other	4.04^a^	1.06, 15.5	4.69	0.92, 23.89	12.29^a^	1.76, 22.83
**Living area**						
Urban area	ref					
Rural area	0.70	0.46, 1.08				
**Source of general vaccination information**						
School (Yes vs. No-ref)			0.33^a^	0.16, 0.69	-5.00	-11.03, 1.03
Magazine/Newspaper (Yes vs. No-ref)	1.49^a^	1.01, 2.20				
Listening to radio or loudspeaker (Yes vs. No-ref)			1.46	0.99, 2.16	3.07^a^	0.03, 6.11
Internet (Yes vs. No-ref)			1.57^a^	1.09, 2.28		
Medical staff (Yes vs. No-ref)			1.43^a^	1.01, 2.01		
Friends and relatives (Yes vs. No-ref)			1.55	0.96, 2.52	3.21	-0.52, 6.94
**Ever heard about HBV vaccine**						
No	ref		ref			
Yes	4.54^a^	1.33, 15.49	1.65	0.79, 3.44		

Abbreviations: Coeff, coefficient; CI, confidence interval; HBV, hepatitis B virus, OR, odds ratio; WTP, willingness to pay.

^a^*P<.05*.

## DISCUSSION

Overall, we found that 62.3% of all study participants were willing to pay for the HBV vaccine, a higher rate than previous studies among Malaysians (37.5%) and Chinese Americans in New York City (53.2%).^18^^,^^19^ Women across the income spectrum were willing to pay for HBV vaccination at similar rates. Among those willing to pay, the mean maximum was US$10.30, over twice the amount found by a similar study in 2016 (108,600 VND, US$4.73).^13^ When participants were asked how much they were willing to pay, differences between income levels emerged for maximum prices greater than US$4.50. We found that 50% of women from Q4 and Q5 were willing to pay a maximum price between US$9–US$18, whereas 50% of women from Q1 were willing to pay less than US$9. When the WTP percentage is increased to 60%, women from Q1 were willing to pay between US$2.30–US$4.50 and Q5 between US$9–US$18.

These data demonstrate that the current market price of the HBV vaccine (approximately US$9) is inaccessible to the majority of low-income women, suggesting the need to subsidize HBV vaccinations for women from low-income households. Ideally, these subsidizations should be funded through national grants, which is typically more stable than foreign aid—though foreign aid may be considered for short-term assistance. However, due to limitations with both of these funding options, a possible solution could be to create a sliding scale for HBV vaccination based on household income in which high-income women pay an amount greater than the market price to subsidize the cost for low-income women. Given that the average maximum price participants were willing to pay was greater than the current market price, it appears that such a vaccination program among WRA could be financed by the high-income recipients of the vaccine alone. However, further research would be required to evaluate consumer acceptance of this payment scheme and to develop an appropriate sliding scale to maximize vaccine uptake.

These data demonstrate that the current market price of the HBV vaccine is inaccessible to the majority of low-income women, suggesting the need to subsidize HBV vaccinations for women from low-income households.

Because HBV is primarily transmitted vertically in Vietnam, we believe that increasing vaccination among WRA would work synergistically with current neonatal vaccination efforts to decrease transmission and confer immunity. This effort is especially important for women in rural or mountainous areas, whereas many as half may deliver their neonates at home,^20^ and neonatal vaccination may not be immediately available. With limited resources to dedicate toward a widespread HBV vaccination campaign, the government should prioritize vaccinating WRA given their high risk of disease transmission. From a cost-effectiveness standpoint, the benefits of vaccinating WRA extend beyond the individual vaccinated to all of her future children as well.

From a cost-effectiveness standpoint, the benefits of vaccinating WRA extends beyond the individual vaccinated to her future children as well.

At the same time, this vaccination strategy should be paired with an education campaign, given that improved knowledge has previously been demonstrated to be associated with higher WTP.^21^ In particular, our data found that women from Q1 had the lowest level of awareness, suggesting that future campaigns should especially focus on low-income women. Information should be distributed through radio programs and over loudspeakers, which are more readily available to all women. Indeed, we found that mediums requiring greater financial investment, such as television, magazines/newspapers, and the Internet, were significantly less used among women in the lowest-income quintiles. Moreover, some studies have found a higher WTP for vaccines against chronic disease with high morbidity and mortality,^22^ suggesting that future education campaigns should not only focus on raising awareness of HBV vaccination but also provide further education on HBV’s related morbidity and mortality.

The cost of treating HBV and its complications in Vietnam is estimated to total US$4.4 billion in 2008 alone.^23^ Given that HBV prevalence is projected to increase from 6.4 million cases in 1990 to 8.0 million in 2025,^24^ this amount will likely be greater in the coming years. The cost-effectiveness of universal newborn HBV vaccination has been well-studied,^25^ and our proposed strategy would operate synergistically to reduce vertical transmission to neonates at a minimal cost to the Vietnamese government. Aside from an initial investment in research, implementation, and education, this vaccination program would be financially self-sustaining, a step toward more equitable health outcomes for decades to come.

### Limitations

Limitations of this study include its cross-sectional nature, which only allowed for us to test for association without insight into causative relationships—though we were able to correlate some results with a previous study conducted in the same districts in 2016. Another limitation is that this study involves self-reporting, which could predispose participants to recall or social desirability bias. Moreover, while participants do not represent all WRA in Vietnam, we did implement random sampling from both urban and rural environments, ensuring that participants from a variety of backgrounds were included in this survey.

## Notes

**Peer Reviewed**
